# *Malassezia* Yeasts in Veterinary Dermatology: An Updated Overview

**DOI:** 10.3389/fcimb.2020.00079

**Published:** 2020-02-28

**Authors:** Jacques Guillot, Ross Bond

**Affiliations:** ^1^École Nationale Vétérinaire d'Alfort, BioPôle Alfort, EA Dynamyc, UPEC, EnvA, Maisons-Alfort, France; ^2^Department of Clinical Sciences and Services, Royal Veterinary College, Hatfield, United Kingdom

**Keywords:** *Malassezia*, dog, cat, diagnosis, treatment

## Abstract

Lipophilic yeasts of the genus *Malassezia* are important skin commensals and opportunistic skin pathogens in a variety of animals. The species *M. pachydermatis* was first isolated from the skin of a captive Indian rhinoceros with an exfoliative dermatitis in 1925, recognized as an important otic pathogen of dogs in the 1950's, and finally accepted, after several years of controversy, as a common cause of canine dermatitis in the 1990's. Since then, there has been considerable research into the biology of *Malassezia* yeasts and their interaction with their animal hosts. In dogs and cats, *M. pachydermatis* is associated with ceruminous otitis externa and a “seborrhoeic” dermatitis, wherein pruritic, erythematous skin lesions, often with brown/black greasy, malodourous material matting hairs, preferentially develop in intertriginous areas. Skin disease is favored by folds, underlying hypersensitivity disorders, endocrinopathies, defects of cornification, and in cats, various visceral paraneoplastic syndromes. Diagnosis is based on detecting the yeast in compatible skin lesions, usually by cytology, and observing a clinical and mycological response to therapy. Treatment normally comprises topical or systemic azole therapy, often with miconazole—chlorhexidine shampoos or oral itraconazole or ketoconazole. Management of concurrent diseases is important to minimize relapses. Historically, wild-type *Malassezia* isolates from dogs and cats were typically susceptible to azoles, with the exception of fluconazole, but emerging azole resistance in field strains has recently been associated with either mutations or quadruplication of the *ERG11* gene. These observations have prompted increased interest in alternative topical antifungal drugs, such as chlorhexidine, and various essential oils. Further clinical trials are awaited with interest.

## Introduction

The genus *Malassezia* is comprised of a group of lipophilic yeasts that have evolved as skin commensals and opportunistic cutaneous pathogens in a variety of mammals and birds (Guého-Kellerman et al., 2010). The transition from commensal to pathogen is frequent in dogs in particular, and in cats to a lesser extent, such that cases of *Malassezia* otitis externa and *Malassezia* dermatitis are commonly presented to veterinarians in small animal practice (Moraru et al., 2019). For example, the prevalence of otitis externa amongst dogs presenting to primary care practices is around 10% (O'neill et al., 2014), and up to 70% of such cases may be associated with *M. pachydermatis* (Forster et al., 2018). These cases are seldom straightforward to manage, because clinical disease often reflects yeast proliferation due to a disturbance in the normal homeostatic balance of host immunity, on the one hand, and yeast virulence, on the other (Ashbee and Bond, 2010). Thus, successful case management is often dependent upon both treating yeast (and any concurrent bacterial) overgrowth with topical or systemic antimicrobial treatments, as well as identifying and correcting where possible, predisposing factors. Commonly identified factors include concurrent allergic or endocrine skin disease, defects in cornification, or anatomical defects such as skin folds or stenosed ear canals (Bond et al., 2010). By contrast, there are only sporadic reports of *Malassezia* associated with skin disease in large animal species. For example, *Malassezia* overgrowth in the intermammary region and preputial fossa has been implicated in tail-head pruritus and localized dermatitis in horses (White et al., 2006). Goats may present with *Malassezia-*associated seborrheic dermatitis (Pin, 2004; Eguchi-Coe et al., 2011). *Malassezia* otitis has been reported in fennec foxes (Guillot et al., 1994), ferrets (Dinsdale and Rest, 1995), pigs (Pinter et al., 2002), and camels (Kuttin and Glas, 1985).

Recently, the World Association of Veterinary Dermatology commissioned the development of clinical consensus guidelines for the diagnosis and treatment of *Malassezia* dermatitis in dogs and cats (Bond et al., 2020). A panel of specialists/diplomates in veterinary dermatology and mycology prepared a detailed literature review of publications up to mid 2018, and made recommendations on selected topics. The draft document was presented at international veterinary meetings and uploaded on the WAVD website for comment for a period of 6 months. The final version comprised a systematic review of published therapeutic studies, and current information on the ecology, pathophysiology, diagnosis, and prevention of skin diseases associated with *Malassezia* yeasts in dogs and cats.

In view of the free access availability of this recent very detailed and wide-ranging review (Bond et al., 2020), the present article aims to provide the reader with a general background summary of current understanding of the roles of *Malassezia* yeasts in animal skin disease, with emphasis on recent publications from mid 2018 to end of 2019 that expand upon previous knowledge.

## The Genus *Malassezia*: A Growing Number of Species in a Growing Number of Animal Hosts

Originally thought as a single species, *Malassezia* yeasts are now known to form a unique cluster consisting of 18 species living almost exclusively on the skin and mucosal sites of warm-blooded vertebrates (Lorch et al., 2018; Theelen et al., 2018) (Table 1). During the last decade, the analysis of the genome of *Malassezia* yeasts suggested that their ancestors were plant or soil fungal residents which progressively managed to survive and develop in the cutaneous ecosystem (Xu et al., 2007). The genus *Malassezia* (Baillon) was created in 1889 for a single species, *M. furfur*, detected in cutaneous lesions in humans (Baillon, 1889). Weidman was the first scientist to detect *Malassezia* yeasts from the skin of an animal, an Indian rhinoceros (*Rhinoceros unicornis*) with a generalized exfoliative dermatitis (Weidman, 1925). In contrast to *M. furfur* these yeasts cultivated readily on routine media without lipid supplementation. *Malassezia* yeasts were further detected from different warm-blooded vertebrates and several specific names were proposed according to the latin names of the animals on which the yeasts were initially isolated: *M. pachydermatis* (from a rhinoceros within Pachydermata, an obsolete nineteenth-century taxonomic order of mammals) (Weidman, 1925), *M. caprae* from goats (Cabanes et al., 2007), *M. equina* from horses (Cabanes et al., 2007), *M. cuniculi* from rabbits (Cabanes et al., 2011), *M. pstittaci* from parrots (Cabanes et al., 2016), and very recently *M. vespertilionis* from bats (Lorch et al., 2018). Some *Malassezia* yeasts (especially *M. pachydermatis*) appear to have a broad host range, while others are more host-specific with a close adaptation to the cutaneous ecosystem of a single animal species or a group of phylogenetically related animals (Table 1). The number of currently described *Malassezia* species (*n* = 18) is likely limited due to a sampling bias toward humans and domestic animals. We can imagine that the number of *Malassezia* species will increase when the skin microbiota of a broader range of wild animals is investigated.

**Table 1:** *Malassezia* species and main mammalian hosts.

***Malassezia* species**	**Synonyms**	**Presence on healthy skin**	**Presence in lesions**
*M. furfur*	*Pityrosporum ovale*	In humans Sometimes in animals	In humans (PV, FG)
*M. pachydermatis*	*P. pachydermatis*,*P. canis*	In dogs, cats, many others (mostly canids) Sometimes in humans (dog contact)	In dogs, cats, others (SD, OT) Sometimes in humans (FG)
*M. sympodialis*	*M. furfur* serovar A	In humans and animals	In humans (AD, SD) Sometimes in cats (OT)
*M. globosa*	*P. orbiculare* *M. furfur* serovar B	In humans and animals	In humans (PV, SD, AD) Sometimes in cats (OT)
*M. obtusa*		In humans	In humans
*M. slooffiae*		In pigs, cats (claws) In humans	In humans
*M. restricta*	*M. furfur* serovar C	In humans	In humans (SD)
*M. dermatis*		In humans	In humans (AD)
*M. japonica*		In humans	In humans (AD, SD)
*M. nana*		In cats, horses	In cats, cattle (OT)
*M. yamatoensis*		In humans	In humans (SD)
*M. caprae*		In goats	
*M. equina*	*M. equi*	In horses	In horses
*M. cuniculi*		In rabbits	
*M. arunalokei*		In humans	In humans
*M. brasiliensis*		In parrots	–
*M. psittaci*		In parrots	–
*M. vespertilionis*		In hibernating bats	–

–, *not reported; PV, pityriasis versicolor; FG, fungaemia; AD, atopic dermatitis; SD, seborrheoic dermatitis; OT, otitis*.

Very recently, Lorch et al. were able to isolate a new *Malassezia* species from the skin of nine species of bats in the subfamily *Myotinae* in eastern and western United States (Lorch et al., 2018). Physiological features and molecular characterization at seven additional loci clearly demonstrated that all of the bat *Malassezia* isolates represented a single and new species which was designated as *M. vespertilionis*. Among other characteristics, the new species is able to grow over a broad range of temperatures (7–40°C), with optimal growth occurring at 24°C. The authors suggested that the wide thermal growth range may be an adaptation to survival on bat skin during both hibernation and active seasons (Lorch et al., 2018).

## Recent Re-assessments of Lipid-Dependence in *Malassezia pachydermatis*

*Malassezia* species are lipid dependent due to an inability to synthesize long-chained (C14 or C16) fatty acids *de novo* (Shifrine and Marr, 1963). There are some differences in lipid dependence among the species and this variability has been used for the development of specific tests for their identification (Guillot et al., 1996). Historically *M. pachydermatis* was regarded as being “lipophilic but not lipid-dependent” because it was the only member of the genus to grow on Sabouraud's dextrose agar (Guillot and Bond, 1999). Recently, genome sequencing has confirmed that *M. pachydermatis* lacks a fatty acid synthase gene like the other members of the genus (Wu et al., 2015), but is uniquely able to utilize lipid fractions within the peptone component of Sabouraud's dextrose agar for growth (Puig et al., 2017). These observations explain its failure to grow on lipid-free defined media and thus *M. pachydermatis* should now also be regarded as being “lipid-dependent” (Puig et al., 2017).

## Ecology of *Malassezia* Yeasts in Dogs and Cats: Complementary and Conflicting Results from Traditionally Culture-Based Studies and More Recent Molecular Investigations

To better understand the ecology of *Malassezia* yeasts on healthy skin and in cases of cutaneous lesions, culture-based studies have been carried out both in humans (Gaitanis et al., 2012) and in animals, especially in dogs (reviewed by Bond et al., 2020). Results vary between studies because of the use of different sampling procedures, culture media, and identification techniques. However, culture-based studies clearly demonstrate that *M. pachydermatis* is the predominant cutaneous yeast in both healthy dogs and dogs with *Malassezia* dermatitis or otitis (Gustafson, 1955; Dufait, 1985; Hajsig et al., 1985; Bond and Lloyd, 1997). *M. pachydermatis* is also most important in cats but in this host other *Malassezia* species are more frequently detected (Hajsig et al., 1990; Hirai et al., 2004; Åhman et al., 2007a; Åhman and Bergstrom, 2009; Volk et al., 2010). Several investigators explored *Malassezia* colonization in various anatomical regions of different breeds of adult healthy dogs (reviewed by Bond et al., 2020). The general conclusion from these studies is that the peri-oral region and interdigital skin is frequently colonized (up to 80%) by *M. pachydermatis* in healthy dogs of various breeds, whereas the yeast is less-often (<25%) detected on the skin of the axilla, groin and dorsum. The skin of cats may be colonized by several *Malassezia* species. Whilst *M. pachydermatis* remains most prevalent, as in dogs, the lipid-dependent species isolated from cats include *M. sympodialis, M. globosa, M. furfur, M. nana*, and *M. slooffiae* (reviewed by Bond et al., 2020). *M. nana* is the most common lipid-dependent species in cats, particularly in the ear canal, and a particular *M. nana* genotype seem to predominate in this animal host (De Bellis et al., 2009; Castella et al., 2011). *M. slooffiae* is primarily but not exclusively isolated from claw folds in cats (Åhman et al., 2007a).

Recently, methods based on next generation sequencing (NGS) have allowed a better characterization of the complex microbial communities occurring on animal skin and made it possible to detect *Malassezia* species that would otherwise be missed using culture-based methods (Meason-Smith et al., 2017, 2019; Korbelik et al., 2018; Older et al., 2019). Meason-Smith et al. reported that the cutaneous mycobiota in dogs was influenced by various factors including environmental exposure, cohabitation with other pets and skin health status (Meason-Smith et al., 2015). Surprising, *Malassezia* yeasts were not the most abundant fungal organisms on healthy canine skin. Furthermore, these authors were unable to detect any significant differences in the relative abundance of *Malassezia* yeasts between healthy and allergic dogs. The discrepancy between NGS results and culture-dependent studies demonstrating increased populations of *M. pachydermatis* in allergic dogs (Bond et al., 1994; White et al., 1998) may be related to differences in methodology. Another explanation would be that dysbiosis is present at *Malassezia* species level (rather that at *M. pachydermatis* abundance) in allergic dogs. This hypothesis was very recently investigated by Meason-Smith et al. who collected skin samples from healthy, naturally affected allergic, and experimentally sensitized atopic dogs (Meason-Smith et al., 2019). Using NGS (at species level classification) and *Malassezia* species-specific quantitative real-time PCR (qPCR), they demonstrated that *M. globosa* was significantly more abundant on healthy canine skin (by both methods), *M. restricta* was significantly more abundant on healthy skin (by NGS), and *M. pachydermatis* was significantly more abundant on naturally-affected allergic skin (by NGS) and on allergen-induced atopic skin lesions (by qPCR).

The NGS method was also recently applied to better understand the mycobiota in the external ear canal of dogs (Korbelik et al., 2018). Samples were collected from six dogs with otitis externa and five clinically normal dogs. In cases of otitis externa, the mycobiota was largely dominated by *Malassezia* yeasts. Fungal species diversity, richness and evenness were all significantly reduced in samples from otitis externa when compared to healthy ears.

In cats, metagenomic analyses suggested that the skin is inhabited by bacterial communities that are distinct to each body site (Older et al., 2017) whereas fungal communities seem more unique to the individual level (Meason-Smith et al., 2017). When samples from healthy and allergic cats were collected, the most abundant fungal sequences were identified as filamentous contaminants from the environment and not *Malassezia* yeasts, which were identified in 30 and 21% of healthy and allergic cat samples, but rarely accounted for more than 1% of the relative fungal abundance (Meason-Smith et al., 2017). The objectives of the recent study from Older et al. were to evaluate how genotype and environment can influence the bacterial and fungal microbiota of feline skin (Older et al., 2019). Using NGS and *Malassezia* qPCR, they demonstrated that *M. restricta* and *M. globosa* were the most prevalent *Malassezia* species. Sequences corresponding to *M. slooffiae, M. furfur, M. nana, M. pachydermatis, M. dermatis, M. sympodialis, M. japonica, M. obtusa*, and *M. yamatoensis* were also detected. *Malassezia* abundance was significantly different between cat breeds with Devon Rex cats having the highest abundance. No significant difference in abundance of any *Malassezia* species were found between the different cat breeds or when comparing indoor and outdoor animals.

Taken together, these studies demonstrate significant disparity between cultural and molecular studies in defining the *Malassezia* species component of the skin mycobiota in dogs and cats. Importantly in dogs, cultural methods seldom demonstrate *Malassezia* species other than *M. pachydermatis* in both health and disease, even when culture media reputed to support the growth of the more lipid-dependent species such as modified Dixon's agar are utilized (reviewed by Bond et al., 2020). By contrast, molecular techniques indicate the frequent presence of *M. globosa* and *M. restricta*, species that are seldom identified by culture (Meason-Smith et al., 2019), despite years of searching by the present and other authors (Guillot and Bond, 1999). Furthermore, the distinctive morphology of *M. globosa* (Guého-Kellerman et al., 2010) is not systematically observed in clinical cytological specimens from dogs with dermatitis (Bond, R., personal observations). When presented with a clinical case, it may not be strictly necessary for the attending veterinarian to know the species identity of the *Malassezia* causing the disease, provided species variation in drug susceptibility is limited (Tragiannidis et al., 2010). Further developments in both diagnostic and antifungal drug susceptibility testing are urgently required to address these aspects.

## Pathogenesis of *Malassezia* Dermatitis in Dogs and Cats: Background and Recent Advances

There have been significant and recent advances in understanding of the mechanisms of interaction between *Malassezia* yeasts and dogs and cats (reviewed by Bond et al., 2020). The outcome of *Malassezia* growth on the skin (commensal existence or inflammation and disease) is dependent upon the metabolic activities of the yeasts (expression of cell wall and secreted virulence attributes) and the host's innate and adaptive immune defensive responses. Interactions with other skin commensals (especially staphylococci) may also play a role in determining the outcome of colonization in animals, although this area is largely unexplored (Ianiri et al., 2018), especially in dogs and cats. All these processes should ideally result in a delicately balanced homeostatic relationship. The presence of *Malassezia* yeasts within the stratum corneum exposes the host to an array of chemicals, immunogens and allergens, comprising fungal cell wall-associated carbohydrates, proteins and lipids; secreted enzymes that generate both substrates for nutrition, and an array of irritant metabolic by-products (reviewed by Ashbee and Bond, 2010; Sparber and Leibundgut-Landmann, 2017).

In a recent investigation, Czyżewska et al. compared the protein profiles of *M. pachydermatis* isolates from 30 dogs with clinical signs of otitis externa and 34 clinically normal dogs (Czyżewska et al., 2019). The most significant finding was the presence of nicotinamide adenine dinucleotide phosphate (NADP)-dependent mannitol dehydrogenase and ketol-acid reducto isomerase (an enzyme involved in the biosynthesis of branched-chain amino acids) among *M. pachydermatis* isolates obtained from dogs with otitis externa. It is not clear whether these enzymes confer an advantage to the yeast or act as virulence factors (Czyżewska et al., 2019).

*Malassezia* cell wall carbohydrates are recognized as IgE binding epitopes in humans with atopic dermatitis but recent work highlighted their importance in fungal cell recognition by host phagocytic cells. C-type lectins are proteins that bind carbohydrates in a calcium-dependent manner via highly-conserved carbohydrate-recognition domains (Tada et al., 2006). Mincle, a C-type lectin expressed by activated phagocytes that binds glucosyl and mannosyl-glycolipids from *M. pachydermatis* and *M. sympodialis*, selectively recognizes *Malassezia* yeasts but not other fungi (Yamasaki et al., 2009). Recently, van der Peet et al. reported the total synthesis of a complex β-1,2-mannosyloxymannitol glycolipid from *M. pachydermatis*, which was a potent agonist of human Mincle signaling; these observations may have relevance in the further understanding of antifungal immunity (Van Der Peet et al., 2019). Whilst it is intuitive that similar mechanisms may occur in dogs and cats, species-specific studies are required to verify this.

A recent study suggested that *M. pachydermatis* is able to activate the aryl hydrocarbon receptor (AhR), a nuclear receptor and transcriptional regulator with pleiotropic effects that include down-regulation of immune stimulation, modification of melanogenesis and epidermal cell function, and inhibition of antagonistic microbes (Buommino et al., 2018). Since indole production was not detected in a study of 80 *M. pachydermatis* strains from canine otitis externa, AhR activation by *M. pachydermatis* might be associated with the release of compounds other than indolic metabolites (Kiss et al., 1996).

Experimental models have been used to better understand the pathogenesis of *Malassezia* dermatitis. Cutaneous responses to the application of viable and killed “lipid-dependent” *Malassezia* in laboratory animals (guinea pigs, mice, rabbits) generally comprised focal areas of scaling that most often resolve without treatment upon discontinuation of inoculation (Drouhet et al., 1980; Rosenberg et al., 1980; Faergemann and Fredriksson, 1981; Van Cutsem et al., 1990). Similarly, in laboratory beagle dogs, application of *M. pachydermatis* was associated histologically with epidermal hyperplasia, occasionally with parakeratosis, superficial perivascular dermal inflammation with primarily neutrophils and lymphocytes, and sometimes mast cells (but not eosinophils); features were more severe at sites that were occluded (Bond et al., 2004). Histological changes markedly reduced within 7 days of withdrawal of yeast challenge. Inoculation of suspensions of *M. pachydermatis* into the middle ear and dermis of immunosuppressed mice led to transient infection that resolved within 21 days (Schlemmer et al., 2018). Recently, Merkel et al. developed a minihost (invertebrate) experimental model wherein the pathogenicity of *M. pachydermatis* was evaluated in wild-type (WT) and Toll-deficient *Drosophila melanogaster*. WT flies were resistant to the infection, whereas Toll-deficient flies showed inoculum-dependent mortality rates. Experimental models may prove valuable in the further elucidation of both yeast virulence and host immune factors that are important in disease processes in various species.

The presence of *Malassezia* yeasts on the skin, both in normal and excessive numbers, is known to activate the skin immune system in dogs and cats. *Malassezia* antigens can stimulate innate, antibody and cell mediated immune responses, as well as triggering hypersensitivity reactions (Bond et al., 2010). In animals in which an overgrowth of organisms has occurred, or in individuals that are predisposed to allergic sensitization, the ensuing inflammatory response can lead to clinical signs such as dermatitis and pruritus. Elevated IgE levels to *Malassezia* yeasts or *Staphylococcus* bacteria in human atopic dermatitis are related to the skin severity index. To assess whether a similar association occurs in dogs, Khantavee et al. investigated levels of allergen-specific IgE, IgG1, and IgG2 directed against *M. pachydermatis* and *S. pseudintermedius*, with total IgG levels, and correlated them with lesion severity in dogs with atopic dermatitis (Khantavee et al., 2019). They reported that specific IgE and total IgG against yeasts and bacteria were significantly increased in atopic dogs of all ages. However, no significant relationships were found between the clinical score and any specific immunoglobulin levels for both microbe types.

## Predisposing Factors for Skin Disease in Dogs and Cats

Ideally commensal *Malassezia* yeasts behave as “good citizens” and occupy their ecological niche within the “transitional mantel zone” of the epidermal stratum corneum and follicular infundibulae, influenced by host skin and the external environment (Theelen et al., 2018). Normally, continuous interactions with the host immune system will maintain low numbers of the yeast without generating a clinically-appreciable inflammatory response (Bond et al., 2020). It is well-recognized that *Malassezia* dermatitis in dogs and cats is most often associated with concurrent diseases that are likely associated with altered skin immune function and/or changes in the chemical and micro-climatic conditions at the skin surface (Bond et al., 2020). Thus in dogs, hypersensitivity disorders (especially canine atopic dermatitis), defects of cornification and endocrinopathies are frequently recognized as underlying factors that must be corrected or managed as part of the therapeutic programme (Bond et al., 1996b). Skin folds commonly represent an important local factor in favoring overgrowth by *Malassezia* and or bacteria; this likely reflects local climatic differences involving factors such as reduced air movement, increased skin temperature and humidity, retained secretions, and surface frictional trauma (Jenkinson, 1992). Although not specifically studied in the dog, it is also generally recognized that *Malassezia* dermatitis is more common in tropical climates, and during warm, humid months in more temperate latitudes, reflecting external environmental effects on the skin microbiota (Theelen et al., 2018). This factor is well-documented in human medicine, with warm tropical climates favoring high positive culture rates and greater species diversity (Gaitanis et al., 2012; Leong et al., 2019).

Predisposition to *Malassezia* dermatitis in cats parallels the canine situation in many ways with one important exception. Whilst skin folds and allergic diseases are also commonly identified as factors (Ordeix et al., 2007), feline *Malassezia* dermatitis in older cats is occasionally associated with visceral neoplasia, most commonly feline (pancreatic) paraneoplastic alopecia and thymoma-associated exfoliative dermatitis (Forster-Van Hijfte et al., 1997; Mauldin et al., 2002).

Dog breeds identified to be at increased risk of *Malassezia* dermatitis include West Highland white terriers (WHWT), English setters, shih tzus, basset hounds, American cocker spaniels, boxers, dachshunds, poodles, and Australian silky terriers (Mason, 1992; Plant et al., 1992; Bond et al., 1996b; Mauldin et al., 1997). Devon rex and Sphynx cats, but not Cornish rex cats, are prone to high carriage rates of *Malassezia* yeasts (defined by culture) and a generalized seborrhoeic dermatitis that responds to oral itraconazole (Åhman et al., 2007a,b; Åhman and Bergstrom, 2009; Volk et al., 2010). These breed predilections in dogs and cats are likely analogous to the observed effects of ethnicity on *Malassezia* populations on human skin (Leong et al., 2019).

A recent study that utilized next generation sequencing (NGS) to study fungal populations on skin and mucosae in various cat breeds also reported that Devon rex cats had a high abundance of *Malassezia* species (Older et al., 2019). Interestingly, species-level analyses of the sequences identified *M. globosa* and *M. restricta* as the most abundant *Malassezia* species in the subject cats (Older et al., 2019). In contrast, previous cultural studies typically identify *M. pachydermatis* as the most abundant species in cats, even when media (for example, modified Dixon's agar) and temperatures (32–34°C) considered appropriate for the cultivation of the more demanding species are adopted (Bond et al., 1996a, 1997, 2008; Volk et al., 2010). Further investigation of these discordant results is warranted.

## Clinical Presentations in Dogs and Cats

Affected skin is usually erythematous, often with greasy brown-black material matting the lower portion of hairs; intertriginous zones are frequently involved (Bond et al., 2010). Pruritus, whilst ranging from minimal to severe, is normally a dominant feature. Concurrent hyperpigmentation, lichenification, malodour, traumatic alopecia, and otitis externa is common. In otitis externa, the discharge from the ear canal is commonly ceruminous and rarely purulent, and inflammation commonly extends onto the pinnae. Cases of *Malassezia* paronychia present with claw fold erythema and swelling, waxy or crusty brown exudate, red-brown claw staining, and may co-exist with a wider pododermatitis of haired skin. An occasional presentation of frenzied facial pruritus in dogs with varying, sometimes subtle, erythema of chin / perioral skin, may be misdiagnosed as neurological disease (Mason, 1992, 1993).

The signs of *Malassezia* dermatitis may mimic, or complicate, those of canine atopic dermatitis. Features of concurrent diseases may be evident initially although they are commonly best appreciated once secondary *Malassezia* infection is removed. *Malassezia* dermatitis might feature in cats that present with a phenotype of allergic skin disease, idiopathic facial dermatitis (Persian/Himalayan), feline acne, and serious internal medical disorders such as feline paraneoplastic alopecia and thymoma-associated exfoliative dermatitis (Bond et al., 2010). Client expectation should be managed accordingly; residual skin disease commonly remains despite successful antifungal therapy.

## Diagnostic approach in the Veterinary Clinic

Following the original elegant description of tape-stripping in human dermatology (Keddie et al., 1961), this method has gained wide acceptance in veterinary clinical practice as a rapid and versatile method for recovering stratum corneum cells and their attendant adherent microbes (Maynard et al., 2011). Light microscopical examination (40–50 or 100x oil objectives) of tape-strips, or dry scrapes, stained with modified Wright Giemsa stain (“Diff-Quik” or generic equivalents) is rapid and convenient for assessment of the presence and numbers of *Malassezia* yeasts (Moraru et al., 2019). Factors such as important variations in anatomical site, breed, sampling method and host immune status commonly thwart the interpretation of the clinical significance of an observed population (“XX yeasts in YY fields”); consequently a “treat what you see” trial therapy approach with topical or systemic antifungal drugs is routinely required to establish the clinical significance of an observed population. A recent clinical consensus guideline document presents a detailed diagnostic algorithm for use in the veterinary clinic (Bond et al., 2020). The importance for investigating and correcting concurrent skin diseases and other predisposing factors, where possible, cannot be over-emphasized, if a chronic or relapsing course is to be prevented.

Cytology using swabs of lesions rolled onto glass slides is normally best restricted to use in the ear canal, as the yield of squames and yeast from the skin is inferior to that obtained by tape strips and dry scrapes (Bond and Sant, 1993; White et al., 1998; Bensignor and Carlotti, 1999). In a recent randomized, blinded prospective study of 30 dogs with otitis externa, cytological specimens obtained using a conventional cotton-tipped swab contained comparable numbers of yeasts and bacteria, but fewer inflammatory cells, when compared with samples prepared by aspiration of material from the horizontal canal with a soft rubber feeding tube (Choi et al., 2018). In an effort to improve upon the sensitivity of cytological sampling for *M. pachydermatis* in the canine ear, Puig et al. have developed a quantitative PCR method based on amplification of the single copy ß*-tubulin* gene (Puig et al., 2019). The authors judged that the results were accurate and showed improved sensitivity over cytology; this method may have useful applications in diagnosis and therapeutic monitoring, and in studies of pathogenesis and therapeutic product development.

## Antifungal Drug Susceptibility Testing for *M. pachydermatis*

Antimicrobial resistance has emerged globally as a serious threat to human and animal health (Fera et al., 2009). Recent publications (Brilhante et al., 2018; Schlemmer et al., 2019a) support previous observations that most wild-type *Malassezia* yeasts remain susceptible to the commonly-used azole drugs such as itraconazole, ketoconazole and miconazole, although efficacy of fluconazole is more variable (Velegraki et al., 2004; Cafarchia et al., 2012a,b; Weiler et al., 2013). In *M. pachydermatis* isolates from canine otitis externa, synergistic interactions have been reported between between caspofungin and itraconazole or fluconazole (Schlemmer et al., 2019a), whereas amphoterecin B antagonized the activity of itraconazole, but not fluconazole or posaconazole (Alvarez-Perez et al., 2019).

In view of routine susceptibility and an absence of standard methods appropriate for the *Malassezia* genus, diagnostic testing in veterinary practice tends to rely upon cytological rather than cultural methods. However, laboratory studies of *M. pachydermatis* have previously demonstrated that it is possible to select for resistance to terbinafine and azoles (Nakano et al., 2005; Jesus et al., 2011). Of greater concern are recent sporadic reports of therapeutic failure with azoles in canine *M. pachydermatis*-associated dermatitis that were associated with increased azole tolerance *in vitro*; this might reflect the chronic and relapsing course of *Malassezia* dermatitis and otitis that often necessitate frequent and lengthy treatment courses (Chiavassa et al., 2014; Watanabe et al., 2014).

Angileri et al. isolated *M. pachydermatis* from an azole-unresponsive toy poodle that had MICs that were several fold higher when compared with strains from untreated dogs (Angileri et al., 2019). Kano et al. showed that an isolate of *M. pachydermatis* with MICs of itraconazole and ketoconazole of >32 mg/L by Etest had mis-sense mutations in the *ERG11* gene that encodes lanosterol 14 –alpha-demethylase, the target site for antifungal azoles (Kano et al., 2019b). Mutations in the same gene were described in field isolates with tolerance to ravuconazole (Kano et al., 2019a) and in miconazole-resistant clones of CBS1879 (the neotype culture of *M. pachydermatis*) selected by serial passage on miconazole supplemented media (Kano and Kamata, 2019). Azole resistance in *M. pachydermatis* has also been associated with quadruplication of the *ERG11* gene (Kim et al., 2018). By contrast, mutations in drug efflux pumps, a common mechanism of azole resistance in *Candida* species (Sanguinetti et al., 2005), has not yet been reported in the genus *Malassezia*. The emergence of azole resistance amongst *Malassezia* species warrants careful surveillance and product stewardship to ensure ongoing utility of this important drug class. Further data are urgently required to establish whether topical therapies are preferable to systemic treatments in this context, and to guide antimicrobial stewardship policies for antifungal therapy in small animal practice.

Concern over azole resistance has prompted heightened interest in alternative antifungal agents. There are reports of *in vitro* efficacy against *M. pachydermatis* of a honey-based gel (Oliveira et al., 2018), monensin and, to a lesser extent, narasin (polyether ionophores originally marketed as anti-coccidials and growth-promoting modifiers of the bovine rumen flora; Chan et al., 2018, 2019). Multiple recent publications have explored the potential antifungal utility of essential oils, complex mixtures of highly concentrated aromatic oils (primarily terpenes and/or phenylpropanoids) extracted from plants by steam distillation, hydrodiffusion or pressure (Manion and Widder, 2017; Bismark et al., 2019). A previous randomized clinical trial reported persistent efficacy of a commercial essential oil product (Malacalm, Flora Slr Oli essenziali, Lorenzana, Italy) applied twice daily for 1 month to dogs with *Malassezia* dermatitis (Nardoni et al., 2014), although the study is weakened by incomplete data on randomization and clinical scores (Bond et al., 2020). Publications between 2013 and 2018 were usefully reviewed by Donato et al. (2019). Most of the recent studies have been conducted *in vitro* and their utility in clinical practice remains largely untested. Comparisons between studies are hampered by an absence of agreed standard testing methods that are not yet optimized, arbitrary assignment of interpretative criteria, and likely batch variation in activities of essential oils prepared by different methods (Bismark et al., 2019). Recently, anti-*Malassezia* effects have been observed *in vitro* using winter savory, lemon grass, oregano, palmarosa and cinnamon leaf oils by agar disc diffusion and vapor assays (Bismark et al., 2019). Oregano oil and thyme oil and their major phenolic components (carvacrol, thymol) were fungicidal against *M. pachydermatis* when tested using agar dilution (Sim et al., 2019). There are recent reports of synergistic interactions between essential oil components and azoles or nystatin against *M. pachydermatis*, including carvacrol and miconazole or nystatin, thymol and nystatin (Schlemmer et al., 2019b), and between clotrimazole and essential oils of *Melaleuca alternifolia, Mentha piperita*, and *Origanum vulgare* (Bohmova et al., 2019).

## Treatment of *Malassezia* Dermatitis in Dogs

A recent evidence-based review on the treatment of canine *Malassezia* dermatitis reported “strong” evidence for the use of a 2% miconazole and 2% chlorhexidine shampoo, used twice weekly (Bond et al., 1995, 2020; Maynard et al., 2011). “Moderate” evidence was available for a 3% chlorhexidine shampoo (Maynard et al., 2011; Bond et al., 2020). For canine cases where topical therapy is ineffective or impractical, there was “moderate” evidence for the use of ketoconazole at 5–10 mg/kg orally once or twice daily; and itraconazole at 5 mg/kg orally once daily or two consecutive days per week (reviewed by Bond et al., 2020). Recently, the clinical and cytological effects of a once daily application of a leave-on spray formulation containing zinc, ethyl lauroyl arginate, laureth-9, urea, panthenol, glycerine and butylene glycol (Aptus® Derma Spot On Concentrate^TM^, Orion Pharma Animal Health, Sollentuna, Sweden) were evaluated in a randomized, blinded, controlled study of 18 dogs with chronic pododermatitis associated with *Malassezia* yeasts (Sjostrom et al., 2018). When compared with placebo treatment of the contralateral foot, reduced yeast counts from the actively treated foot were associated with a reduction in clinical scores.

In dogs, persistent or recurrent *Malassezia* dermatitis are usually associated with failure to identify and correct predisposing or perpetuating factors. However, the evidence that reduced susceptibility of *M. pachydermatis* to commonly used antifungal drugs may develop under both field and laboratory conditions highlights the need for surveillance and vigilance for the emergence of clinically-relevant resistance. Agreed reference methods to assess antifungal susceptibility of *M. pachydermatis* are required to assist veterinary practitioner for the management of chronic cases.

## Potential Transmission of *Malassezia* Yeasts from Animals to Humans

The zoonotic potential of *Malassezia* yeasts was first defined in the context of a neonatal intensive care unit, where a cluster of low birth weight patients receiving lipid emulsions were colonized by *M. pachydermatis* that was likely introduced on the hands of health care workers transmitted by contact with pet dogs (Chang et al., 1998). Once introduced to a facility, *Malassezia* yeasts can persist on incubator surfaces for prolonged periods of time (Van Belkum et al., 1994). A subsequent case report described a facial granuloma caused by *M. pachydermatis* in a dog owner (Fan et al., 2006), and recently *M. pachydermatis*-associated fungemia has been reported in a small number of adults with various predisposing factors (Choudhury and Marte, 2014; Roman et al., 2016; Lee et al., 2019). Since hand contamination by *M. pachydermatis* is common amongst dog owners, especially in owners of allergic dogs with *Malassezia* overgrowth (Morris, 2005), there is a clear need for rigorous hand hygiene by individuals in contact with pet dogs and cats, especially when there is contact with immunocompromised individuals (Bond et al., 2020).

## Author Contributions

JG and RB wrote, revised, edited and approved this manuscript for submission in a collaborative process, agreed on the allocation of the review of these papers by topic, and circulated their draft reviews for the other author to edit and revise as appropriate. RB performed an initial electronic search for recent relevant publications and led the development of the remaining sections. JG led the development of the sections on the genus, ecology, and pathogenesis and uploaded the manuscript.

### Conflict of Interest

In the past 5 years, JG has received funding from or otherwise collaborated with MSD Animal Health, Ceva Animal Health, Bayer Animal Health and Boehringer-Ingelheim. RB has received funding from or otherwise collaborated with Dechra Veterinary Products, Bayer Animal Health, Ceva Animal Health, MSD Animal Health and Elanco.
